# Asthma beliefs among mothers and children from different ethnic origins living in Amsterdam, the Netherlands

**DOI:** 10.1186/1471-2458-8-380

**Published:** 2008-11-03

**Authors:** QM van Dellen, WMC van Aalderen, PJE Bindels, FG Öry, J Bruil, K Stronks, PEACE study group

**Affiliations:** 1 Department of Paediatric Pulmonology, Emma Children's Hospital, Academic Medical Centre, University of Amsterdam, Meibergdreef 9, 1105 AZ, Amsterdam, the Netherlands; 2 Department of General Practice, Academic Medical Centre, University of Amsterdam Meibergdreef 9, 1105 AZ, Amsterdam, the Netherlands; 3 TNO Quality of Life, Division Prevention and Health, P.O. Box 2215, 2301 CE, Leiden, the Netherlands; 4 Department of Social Medicine, Academic Medical Centre, University of Amsterdam Meibergdreef 9, 1105 AZ, Amsterdam, the Netherlands

## Abstract

**Background:**

Doctors and patients hold varying beliefs concerning illness and treatment. Patients' and families' explanatory models (EMs) vary according to personality and sociocultural factors. In a multi-ethnic society, it is becoming increasingly significant that doctors understand the different beliefs of their patients in order to improve patient/doctor communication as well as patient adherence to treatment.

**Methods:**

Twelve focus groups were formed, consisting of 40 children diagnosed with asthma, as well as 28 mothers of these children. These groups included mothers and children of different ethnicities who were living in Amsterdam, the Netherlands. In order to understand the beliefs that both mothers and children hold regarding asthma and its treatment, the explanatory models were analysed and compared.

**Results:**

Study findings show that mothers and children, regardless of ethnicity and age, have their own EMs. Overall, there is a great deal of uncertainty related to the causes, consequences, problems, and symptoms of asthma and its treatment. It also seems that many concerns and feelings of discomfort are the result of lack of knowledge. For instance, the fact that asthma is not seen as a chronic disease requiring daily intake of an inhaled corticosteroid, but rather as an acute phenomenon triggered by various factors, may be very relevant for clinical practice. This particular belief might suggest an explanation for non-adherent behaviour.

**Conclusion:**

A thorough understanding of the mothers' and children's beliefs regarding the illness and its treatment is an important aspect in the management of asthma. Gaining an understanding of these beliefs will provide a foundation for a solid clinician-patient/family partnership in asthma care. Although ethnic differences were observed, the similarities between the mothers' and children's beliefs in this multi-ethnic population were striking. In particular, a common belief is that asthma is considered an acute rather than a chronic condition. In addition, there is a lack of knowledge about the course and the self-management of asthma. Health care providers should be aware of these commonly held beliefs, and this information could be shared in educational programs.

## Background

Asthma is the most prevalent chronic disease among children in Western countries.[1] Despite the availability of effective treatments aimed at controlling asthma symptoms, up to 50% of children with asthma continue to have frequent and bothersome symptoms.[2] US and UK studies among adults and children with asthma suggest that factors such as individual beliefs, attitudes, behaviour, inadequate patient-clinician communication and a foreign ethnic origin may all contribute to poor adherence to treatment with inhaled corticosteroids (ICSs), which in turn results in poor asthma control. [3-7]

As a result of migration, Western European countries are becoming more ethnically diverse. In the Netherlands approximately 10% of the population is of non-Western origin.[8] A logical consequence of this trend is that the number of non-Western children is also growing. In the Netherlands' larger cities, these non-Western children constitute more than 50% of the population.[9] (see after Introduction for more information) Although there is limited literature on asthma prevalence among the Netherlands' largest ethnic minority groups, the number of ethnic children diagnosed with asthma seems to be similar to that among ethnic Dutch children.[10] However, there is evidence that children from ethnic minorities have less control over their asthma.[11]

According to the theoretical model of the medical anthropologist Kleinman, healthcare providers and patients have different beliefs about the patients' illness and treatment. [12,13] Patients' and families' explanatory models (EMs) vary according to personality and sociocultural factors. Studies among ethnic minority populations in the US suggest that cultural factors have an impact on the manner in which patients and families from these groups explain asthma. [14,15] Therefore, it is becoming increasingly recognized that doctors must understand the different beliefs of their patients in order to improve communication as well as patient adherence to treatment. [16,17]

In Europe, there are few studies that describe the differences in beliefs about asthma and its treatment among mothers and their asthmatic children from different ethnic origins. This study explored and compared EMs in *aetiology of the condition*, *onset of symptoms*, *course of illness *and *treatment *of the mothers and children from Moroccan, Turkish, Surinamese and Dutch backgrounds. The purpose of this exploration and comparison was to gain more insight into the groups' beliefs about causes, consequences, symptoms and the self-management of asthma. To elicit the EMs of Moroccan, Turkish, Surinamese and ethnic Dutch mothers and children, a qualitative study was conducted using focus group interviews. Attention was given to the similarities and differences between mothers and children and also among the different ethnicities. The results of this study provide data that can be used in the development of a questionnaire and information aids. These various tools can then be implemented by the clinician when focusing on asthma care and self-management when dealing with the largest ethnic groups in the Netherlands.

### Background of Moroccan, Surinamese and Turkish migrant populations

The Moroccan, Surinamese and Turkish populations are three of the largest ethnic minority groups in the Netherlands. Of the country's approximately 16 million residents, 315,821 are Moroccan, 329,430 Surinamese and 358,846 Turkish.[9] First-generation Turks and Moroccans are mainly economic migrants with a low level of education. [18,19] They came to the Netherlands mainly between 1960 and 1980. The Surinamese group is from the former Dutch colony of Surinam, and consists largely of people of Creole (45%) and Hindustani ethnic backgrounds (45%).[20] Creole people have a mixed background (African and European) and Hindustani people originate from India. The migration to the Netherlands peaked in 1975 when Surinam became independent.[21] With increasing migration, the socioeconomic position of the Surinamese migrant population became more diverse. Of the Turks and Moroccans aged 35 to 54, 74% and 84% respectively have only primary education and there is a higher percentage of illiteracy among Moroccans than among Turks. Of the Surinamese aged 35 to 54, 31% have primary education only. A relatively high percentage of these migrants are unemployed.[22] As a consequence, household incomes and socioeconomic status of the three migrant groups are lower than that of the ethnic Dutch population. However, the Surinamese have a higher socioeconomic status than Turkish and Moroccan migrants.[22] Generally, the Surinamese speak fluent Dutch, mainly because of their colonial past, whereas most Turks and Moroccans have difficulty in speaking Dutch [18,19]. Furthermore, all first-generation migrants remain mainly oriented towards their own ethnic group, [18,19,21] but the Surinamese have relatively more contact with the ethnic Dutch than Moroccan and Turkish migrants. This may be explained in part by their familiarity with the Dutch language and culture.[21]

## Methods

### Subjects and recruitment

Children and mothers only were recruited by one physician (first author QvD) who had previously seen all these children and mothers in a home setting as part of a multicentre study. During these home visits, detailed information was collected including the child's age, gender, ethnicity, level of education, use of asthma medication and level of asthma control. Moreover, information about the parental level of education and socioeconomic status was also collected. A detailed description of this study has been previously published.[11] Briefly, the inclusion criteria for children in this multicentre study were: 1) having asthma diagnosed by a paediatrician, 2) attending an outpatient clinic of one of the six participating hospitals, 3) being aged between 7 and 17 years, 4) being of Moroccan, Turkish, Surinamese or ethnic Dutch descent. Children were classified as belonging to an ethnic group other than Dutch if the child itself, or at least one of its parents, was born outside of the Netherlands. An additional criterion for the focus group interviews was that the children were prescribed inhaled corticosteroid (ICSs) in the year prior to the focus group sessions. An attempt was made to gain a well-balanced representation of the children and mothers of the multicentre study (purposive sampling) for participation in the focus group sessions. This involved ascertaining the level of the mothers' education, the socioeconomic status of the family, as well as the age, gender and level of asthma control in the children. After obtaining the adolescents' and mothers' verbal agreement to participate in the study, a letter with further information was sent, followed by a reminder phone call two days before the sessions took place. The study protocol was approved by the Medical Ethics Committees of all participating centres (Sint Lucas Andreas Ziekenhuis, Amsterdam; Slotervaart Ziekenhuis, Amsterdam; VU University Medical Centre, Amsterdam; Onze Lieve Vrouwe Gasthuis, Amsterdam; Zaans Medisch Centrum, Zaandam).

### Focus groups

After the mothers had been subdivided according to ethnicity (ethnic Dutch, Turkish, Surinamese, Moroccan), and the children on the basis of ethnicity and age (6 to 12 year olds (young children) and 13 to 17 year olds (adolescents)), 4 focus group sessions were established among young children (total of 26 children), 4 sessions among adolescents (total of 14 children), and 4 sessions among mothers (total of 28 women). Each session took about two hours and was chaired by two experienced individuals, the moderator and the observer, of the same ethnic background as the participants. Consequently the principal researcher, also first author (QvD), was not present at al sessions. Corresponding ethnic backgrounds enabled the study participants to speak in their native languages and therefore culturally sensitive information was conveyed accurately and sensitively.[23] Each session began with an explanation of the purpose of the focus groups. The moderator followed a semi-structured interview procedure and used a checklist containing questions/topics to be discussed. The observer, who did not actively participate in the interviews, took field notes. The topic guide was based on Kleinman's Explanatory Models.[24] Interviews based on this model focus on five issues: *aetiology of the condition*, *onset of symptoms*, *pathophysiology*, *course of illness *and *treatment*. Examples of questions that were posed to start the discussion include the following: "What is asthma?", "How do you treat asthma?", "Will you or your child be bothered by asthma in the future?", "When does your child take his medication?", "How do you or does your child recognize an asthma exacerbation?" "What do you or does your child do in case of an exacerbation?" In an attempt to promote an informative discussion, open-ended questions were asked as much as possible. However, due to the fact that both the mothers and children sometimes found it difficult to engage in conversation, it became necessary to stimulate and guide them using yes or no questions and examples, such as, "In case of an exacerbation, do you start with salbutamol?", "Do you know that the best way to treat asthma is to use your ICSs daily?", "Do you think a good relationship with your doctor will have a positive influence on the course of asthma?" Furthermore, at the end of each discussion a summary was made by the moderator. Participants were then asked if this summary was accurate and complete, and when necessary, participants provided additional information. All mothers and children were aware that the data would be used in research publications and that the anonymity of the participants would be strictly preserved. Written informed consent was obtained from all children and mothers.

### Analysis

Each focus group was tape-recorded. All recordings were transcribed verbatim by the observer. The moderator also listened to the recordings, then checked, and corrected if necessary, the transcripts. Moroccan and Turkish transcripts were translated by the bilingual observer into Dutch and checked again by the bilingual moderator. The Turkish and Moroccan recordings and transcripts were also checked by bilingual medical students of the same ethnic background. All disagreements were discussed in order to form a consensus. The interviewers' comments and questions, conversation fillers and redundant comments were also part of the translation. Then the data were analysed with the help of the theory of Kleinman. This involved first examining the transcripts and using descriptive codes to compare and look for common patterns and themes within and among the different groups. In other words, each focus group's transcript was systemically read several times by the first author (QvD) and again independently by 7 different experts in the field. Among them the authors FGÖ, JB and KS. Next fragments (or given examples) in the transcriptions were coded and categorized independently. After that the coded fragments, containing the participants' ideas about 'aetiology of the condition', 'onset of symptoms', 'course of illness' and 'treatment' in accordance with the theoretical model of Kleinman were selected from each session and categorised. The category of pathophysiology was explored superficially and therefore omitted from the analysis. Once the coding and categorizing was completed the researchers compared their work and discussed any disagreements in order to form a consensus. A final selection of quotations which seemed to illustrate key issues in each category was made, and this is reproduced in the result section.

## Results

### Characteristics of the participants

Table 1 and 2 show demographics and characteristics of the respectively participating children and mothers. Initially 43 mothers were invited to participate, and 28 (65%) ultimately attended. Forty young children aged 6 to 12 were invited, and 26 attended (65%). Twenty-four older children aged 13 to 17 were invited and 14 attended (58%). Most of those who declined stated they were too ill or lived too far away and did not have any transport. Almost 50% of the participating young children had well controlled asthma. (Table 1) Two of the younger children had recently stopped using their ICSs and two others had stopped using their bronchodilators. There were 14 adolescents participating in the study and all of them were boys. Out of those 14 boys, 43% had well controlled asthma. (Table 1) The 28 mothers involved in the focus groups had in total 32 children. (Table 2) The educational level of most of the mothers was low: 43% had never attended school or had primary education only. The characteristics of the children of these participating mothers were similar to these of the participants of the focus groups among young children and adolescents.

**Table 1:** Demographics and characteristics of participating children and adolescents

**Children/adolescents**	**Dutch**	**Moroccan**	**Turkish**	**Surinamese**	**Total**
**Total number of participating young children (7–12 years)**	5	10	6	5	26
					
**Gender**					
boys	2	4	4	3	13
girls	3	6	2	2	13
**Medication**					
inhaled corticosteroids	5	9	5	5	24
Bronchodilators	4	9	6	5	24
**Asthma control^¶^**					
well controlled	4	4	3	3	14
not well controlled	1	6	3	2	12
					
**Total number of participating adolescents (13–17 years)**	3	4	3	4	14
					
**Gender**					
boys	3	4	3	4	14
girls	0	0	0	0	0
**Medication**					
inhalation corticosteroids	2	4	3	4	13
bronchodilators	3	4	3	4	14
**Asthma control^¶^**					
well controlled	1	2	1	2	6
not well controlled	2	2	2	2	8

^¶ ^information from results multicentre study

**Table 2:** Demographics and characteristics of participating mothers

**Mothers**	**Dutch**	**Moroccan**	**Turkish**	**Surinamese**	**Total**
**Total number of participants**	4	8	10	6	28
					
**Generation^¶^**					
first	n/a	8	10	4	22
Second		0	0	2	2
**Education^¶^**					
yes	4	4	2	6	16
no or primary education only	0	4	8	0	12
**Occupation^¶^**					
skilled labour	4	2	1	3	10
manual labour	0	4	7	3	14
homemaker (no more information)	0	2	2	0	4
					
**Children of participating mothers**	4	10	10	8	32
					
**Gender**					
boys	2	4	8	6	20
girls	2	6	2	2	12
**Age**					
6–12 years	4	8	7	7	26
13–17 years	0	2	3	1	6
**Medication**					
inhalation corticosteroids	4	9	9	8	30
bronchodilators	3	10	10	8	31
**Asthma control^¶^**					
well controlled	3	3	5	4	15
not well controlled	1	7	5	4	17

^¶ ^information from results multicentre study

### Aetiology

None of the study participants, mothers nor children, actually knew what caused asthma. However, most of the participants had some kind of explanatory model explaining their asthma, ranging from their genes to a broad spectrum of triggers which cause asthma symptoms. It was discovered that most children, regardless of age or ethnicity, were taught about the aetiology of asthma by their GPs or paediatricians and not by their mothers or fathers. However, in spite of the ideas the children expressed about the possible aetiology of asthma, the majority of the children stated they were uncertain about the true aetiology:

*"I don't know why I have asthma?" *(the majority of the children), and *"Why do I have asthma and my friend doesn't?" *(a Dutch boy, 8 years old)

In addition, the majority of the mothers, regardless of ethnicity, were uncertain about the cause of asthma. One of the most striking similarities among the mothers was that they seemed to know that asthma has a genetic component:

" *It runs in the family*." (the majority of the mothers)

Moreover, a remarkable difference was seen in the immigrant Moroccan and Turkish mothers. The most commonly reported EM for the aetiology of asthma held by these mothers was the rainy, damp Dutch climate:

*"When we are in Morocco, my child never has asthma." *(a Moroccan mother), "*We live in a very run-down house and because of the damp climate, the house is also very damp. This is the reason my daughter suffers from asthma." *(a Turkish mother)

These types of comments, most frequently stated by the non-Dutch mothers, are examples that suggest that many of these mothers believed that the onset of asthma was related to a particular trigger. In addition to stating that the cold weather triggered asthma, the mothers mentioned dust, pets, cigarette smoke, playing sports and becoming fatigued as other possible triggers. Related to this, a common notion among all mothers and children was the idea of asthma as an acute rather than a chronic illness:

*"Fortunately it is not present every day. Sometimes, very suddenly, it is there. It depends on what I do*."(a Turkish boy, 16 years old)

In other words, the idea of *no symptoms, no asthma *was frequently heard among all mothers and children:

"*During the summer my child doesn't have asthma." *(a Turkish mother), "*My child only has asthma in her father's house." *(a divorced Dutch mother), "*I only have asthma when I'm at school. It is very dusty at school says my mum!" *(a Turkish boy, 8 years)

### Course of illness

This category describes what the participants think of the general course of asthma. All mothers and children, regardless of ethnicity and age, had individual expectations about the course of the illness. These expectations were mainly focused on the consequences of the asthma in both the near and distant future.

#### Concerns/problems

The majority of the Surinamese mothers had the idea that, although their children would be affected by asthma their entire lives, they could live with it and manage the disease:

"*If you know how to deal with your asthma, you can live with it!" *(a Surinamese mother suffering from asthma herself), *"If my child takes his daily medication, he can live a normal life and will have no problems in the future." *(another Surinamese mother)

However, all ethnic Dutch mothers had strong concerns about their children's future, the level of education they could obtain and their careers. They mentioned that their children miss more school than their peers due to illness, and this could influence their marks. An opinion shared by both mothers and children of non-Dutch origin, and which is somewhat similar to the opinion expressed by the Dutch mothers, is that asthma can be particularly bothersome during the night and because of this the children are often very sleepy in the classroom and have trouble following class lessons. Another similarity that was highlighted during most child and mother sessions, regardless of ethnicity and age, was that children with asthma get tired more quickly and experience more physical constraints than children without asthma:

*"I can't do everything my friends can do. Like swimming or gym in school. Most of the times I stand aside." *(a Moroccan girl, 11 years old), *"It is not that I can't do as much as my friends can do, I just get tired earlier and have to stop the activity." *(a Dutch boy, 15 years old)

Because of these physical restrictions, young children are afraid of being bullied and excluded by their peers:

*"None of the boys in our street ever ask me to join a football game, because they know I have asthma and so I can't run fast." *(a Dutch boy, 11 years old)

On the other hand, there were some younger and older children, regardless of ethnicity, who told us that it could also be to an asthmatic child's advantage to tell friends and classmates about having asthma, because then they could show you consideration:

*"I can't go to my friend's house to play, because they have a cat and I'm allergic to cats. Since my friend knows, we always play at my home." *(a Turkish girl, 12 years old)

The Moroccan and Turkish adolescent boys did not share this view. These boys did not see the benefit of telling their friends they are suffering from asthma:

*"As long as no one can see I have asthma, why should I tell them?!" *(a Moroccan boy, 14 years old)

It remained unclear why exactly the Moroccan and Turkish boys felt this way.

#### Prognosis

A remarkable similarity in all mothers was the concern about the duration of the disease:

*"When will my child get better?" *(the majority of the mothers)

Nevertheless, most Turkish and Surinamese mothers were hopeful for the future. The majority of the participants, including mothers and children, shared the belief that asthma was less severe than in the past. Therefore, they were under the impression that the asthma would continue to improve. Some children were even convinced that they could outgrow their asthma. However, contrary to this hopeful idea was the pessimistic view held by the majority of the mothers and children that asthma would always remain a problem in their lives:

*"Once I heard, I forgot who told me, that asthma is a chronic disease, which means, uuhh I have heard this too, that it will always bother me."*(a Surinamese boy, 15 years old), *"I think I will have asthma my entire life, because I was born with it." *(a Turkish boy, 16 years old)

### Onset of symptoms

This paragraph discusses what mothers and children perceived as provoking symptoms of asthma. When discussing the onset of symptoms of asthma, the mothers mentioned a very broad spectrum of triggers. One of the similarities between the majority of the children and mothers, regardless of age and ethnicity, was that they had clear ideas about which triggers provoke asthmatic symptoms. Some mothers mentioned triggers such as dust, grass, milk, peanuts, trees and animals, which can all be tested for in an allergy test. Other mothers mentioned triggers such as fatigue, crying, laughing, playing (sports), perfume, unpleasant smells, fog, rain, humidity, cold weather, cigarette smoke, cold, or spicy foods. All participants were quite sure of the provoking triggers and the following onset of symptoms:

*"It is something you learn over the years." *(a Dutch boy, 14 years), "*I recognise the symptoms very early, I've learned it over the years. It looks like my child is drowning." *(a Moroccan mother), *"The moment my daughter tells me, puffing and blowing, that she is feeling tired, I know her asthma is bothering her." *(a Dutch mother), *"It always starts in the evening, when he is playing around with his younger brother. I always think he will drop dead. He looks so pale!" *(a Turkish mother), *"Thanks to my maternal instinct, I recognise the symptoms immediately. Sometimes my daughter gets stuck halfway up the stairs. I can see in her eyes that she is having a hard time." *(a Surinamese mother)

An interesting difference between the adolescents boys and younger children was that the adolescents expressed experiencing a feeling of sadness when they feel the onset of symptoms, for instance during a football game or at a school party:

"*I feel like a dope when it starts on the dance floor." *(a Turkish boy, 15 years old), "*For me I feel like a weakling when I need to stop before the game is over. Duhhhh, for me the game is over..." *(a Moroccan boy, 14 years old)

### Treatment

Asthma causes problems in the daily lives of affected children and their families. Treating this disease is a necessity, and the children and their families must find ways to manage the asthma. This section of the paper explains how children and mothers manage the asthma.

#### Avoiding triggers

In all sessions, regardless of ethnicity and age, we saw that children and mothers knew that by avoiding triggers, they could reduce the risk of an asthma attack. But even with this understanding that asthma triggers should be avoided, in actual practice it appeared to be sometimes difficult to do so. In particular, the avoidance of some triggers was considered to be beyond the control of the individual:

"*Sometimes I can't avoid the trigger. I can't help it if it's misty!" *(a Moroccan boy, 14 years old), *"In our culture, it is bad manners to ask a guest not to smoke in the house." *(Moroccan and Turkish mothers), *"My child needs anti-allergic bed covers, but my insurance company will not reimburse the costs, so I can't afford it." *(a Moroccan mother), "*I don't want to stay home when my friends are going to the school party. But I can't avoid it when they smoke." *(a Dutch boy, 15 years old)*, "Sometimes I feel confused. The doctors and my mum tell me it is good and healthy to do some sports. But, on the other hand, when I start running I'm short of breath." *(a Surinamese girl, 12 years old)

In addition to being aware of the importance of avoiding triggers, a clear similarity among all mothers, regardless of ethnicity, was the idea that their children should live healthy lives in order to suffer fewer side effects from asthma. During all focus group interviews, both mothers and children mentioned that clean houses, no pets, healthy food, no smoking and plenty of exercise are factors which will help reduce asthma symptoms.

#### Medication

In all sessions, regardless of age and ethnicity, mothers reported that two forms of management include the daily ICSs and remembering to take salbutamol with you when leaving the house. When we asked more specifically about the proper use of ICSs as maintenance therapy, all mothers, with the exception of the Surinamese, expressed reservations in some form or another. Their experience with the efficacy of ICSs as maintenance therapy was very good. The Surinamese mothers even mentioned that they supervised the use of medication, in order to make sure that their children inhaled the right medication at the right time. Most Moroccan mothers voiced that they performed a self-assessment, and also their assessment was more emotionally then rationally supported:

"*If my daughter does not show any signs of asthma, I can't give her uuhh, her orange medication. I think it's very sad for children to take daily medication when they are feeling OK." *(a Moroccan mother)

The Dutch mothers shared an overall negative attitude towards ICSs. Most Dutch mothers reported performing a detailed self-assessment of the state of their childrens' asthma before deciding whether or not to administer their ICSs. They mentioned this is something they have learned over the years. Most of the time these decisions conflicted with their doctors' instructions. Some of the children were taken off ICSs by their mothers when symptoms were absent, and other mothers terminated therapy when symptoms did not abate despite adherence:

"*Often I do not give my child her Seretide, because I fail to see the point of it. Treatment based on maternal instinct is still the best"*. (a Dutch mother)

This statement was based on the mother's maternal instinct and feeling rather than knowledge. Another reason behind their decisions to sometimes not give the medication was that the Dutch mothers also worry about long-term side effects of ICSs. These mothers thought it would be harmful to give them for a long period. The Turkish mothers were also under the impression that the use of ICSs will have some side effects. The most frequently mentioned side effects, which were also mentioned as the major factor preventing mothers from giving children their ICSs, were weight gain, growth retardation and addiction.

The only mothers who mentioned using alternative medication with their children were the Turkish and Dutch mothers. When the Turkish mothers administered alternative medication to their children, they stopped the regular medication. The Dutch mothers, however, mentioned using a combination of alternative and regular medication. Examples of the alternative medication used includes over – the-counter drugs and homemade herbal (cough) mixtures.

We also observed reservations towards medication among adolescents. The majority of the adolescents admitted they listen to their bodies when deciding whether to take their ICSs, although their mothers remind them daily:

*"It's rather annoying my mother asking me daily if I've taken my Flixotide. What's that got to do with her? I don't want to get mixed up in her problems either. I'll take it when I feel symptoms." *(a Turkish boy, 15 years old), *"I manage my asthma myself. I'm old enough and wise enough to do so!" *(a Moroccan boy, 15 years old)

In contrast, the majority of the younger children, regardless of ethnicity, were not reluctant to take their ICSs. However, almost all the children mentioned forgetting to take their ICSs on a daily basis. The majority of the children expressed that a reason for taking their ICSs daily is because their parents tell them to, not because they are aware of the effects of ICSs. While most children are reminded by their parents, others put their medication in places where it will catch their eye, for instance on the TV remote control or on the computer.

#### Relationship with doctors

Another important element in the management of asthma, according to all the mothers and children, is their relationship with their doctors. All mothers and children expressed that they had a fairly good relationship with their paediatricians, but less so with their general practitioners (GPs). The most frequently heard reasons for being satisfied with the care were the doctor taking enough time for the visit, listening to the complaint and taking it seriously, and being easily accessible and friendly. Many Moroccan and Turkish mothers said that they experienced a linguistic barrier and found it difficult to make themselves clear. One of the most frequently heard complaints among children as well as mothers was that their doctor could be very impersonal and cold:

"*He only looks at his computer, instead of looking at my son. As if the computer is his patient!" *(a Moroccan mother)

## Discussion

To our knowledge, this study provides some of the first published data in Europe focusing on the beliefs of mothers and children from different ethnic backgrounds related to asthma and its therapy. More specifically, we tried to understand and explain their Explanatory Models regarding asthma. These proved to be broadly similar across ethnic groups, although there were some variabilities in beliefs regarding asthma and its treatment by ethnicity and also by age group. For instance, when focusing on the aetiology, we found that Moroccan and Turkish mothers in our study were more likely than ethnic Dutch and Surinamese mothers to mention that the Dutch climate caused their children's asthma. Moreover, a consequence of the damp Dutch climate is that most of the inexpensive, run-down houses are also very damp. A recent study showed a consistent association between reported moulds and dampness in the living room or the child's bedroom and an increased risk for severe airway hyper responsiveness.[25] Since most of the immigrant Moroccan and Turkish people living in Amsterdam live in these run-down, damp houses,[26] it is not surprising they expressed this to be the cause of asthma in their children. In relation to this, many of these mothers indicated that as long their children live in the Netherlands, their children will always suffer from asthma. Other non-Dutch mothers share the belief that it is impossible for their children to one day live without asthma as the triggers are always present.

There were few differences between the ethnic and age groups with regard to the beliefs towards the onset of symptoms. Our study results showed that all mothers were extremely aware of the triggers which cause asthma symptoms in their children, as well as signs of an asthma exacerbation. Two factors appeared to be especially important for good asthma management: (1) parental support, and (2) putting the asthma medication in a highly visible place which will help the children remember to take their medication. Apart from good management, however, a common belief in all focus groups was that additional adequate care by paediatricians and GPs is important for asthma control. Clinicians must be prepared to work in an ongoing partnership with patients and parents to ensure that they are offered a clear rationale as to why an ICSs is necessary and to address the concerns about potential adverse effects.[27] This approach should be based on a detailed examination of patients' perspectives on asthma and its treatment. In addition, clinicians must treat patients in an open, non-judgemental manner. By improving their understanding of the different beliefs of their patients, clinicians can communicate more effectively and increase patient adherence to treatment. [16,17]

All mothers had doubts about how asthma could interfere with the social and economic future of their children. The Dutch mothers were particularly concerned about this. These mothers, who had the highest socioeconomic status of all the mothers, were worried about the educational possibilities and future careers available for their children who suffered from asthma. In addition, most of the younger children expressed a fear of being excluded by their peers because of their asthma. Conducting long term follow up research with these children may provide interesting results regarding whether having asthma at a young age has a detrimental effect on the children as they get older. Given these worries, it is interesting to note that instead of following the doctors' advice to use ICSs daily, the ethnic Dutch mothers expressed that they make a detailed self-assessment of the state of their children's asthma before deciding whether or not to administer their ICSs.

The topic that highlighted the most significant ethnic differences was treatment. Similar to other studies, we can conclude that the mothers decided to discontinue medication for reasons such as fear of unknown side effects and disbelief in the need for daily therapy. [28-30] Horne and Weinman also suggested that patients will be more likely to adhere to preventive medication if their beliefs in its necessity outweigh their concerns about potential adverse effects.[31] Moreover, we know that in addition to misconceptions, fear of unpleasant side effects or addiction and disbelief, other factors such as unmet expectations, failure by healthcare workers to address health beliefs and patient preferences, and inadequate patient-clinician communication have also been reported to be associated with non-adherence, especially amongst children from ethnic minority groups. [3-7] The Turkish and Moroccan mothers in this study who had difficulties with the Dutch language stated that their relationship with their doctors was fairly good, but they experienced a linguistic barrier and found it difficult to make themselves clear. Therefore, the patient-clinician communication may have been hampered. This in turn could have led to non-adherence to treatment or caused mothers to feel the need to manage the asthma care of their children on their own.

The idea of *no symptoms, no asthma *is a health belief which is seen world wide.[32] We sensed that the majority of our participants, regardless of ethnicity and age, shared this health belief. This brings us to the conclusion that concerning the chronicity of asthma, the participants' EMs differed from the biomedical model of asthma. Our participants also see asthma as a situation that appears and disappears, rather than as a chronic inflammatory condition which needs daily ICSs. This *no symptoms, no asthma *belief could also be an explanation for non-adherence to daily ICSs. [32-34] Moreover, in accordance with previous studies, this belief and the related behaviour (not taking ICSs) was seen more often in adolescents than in young children. From a developmental perspective, adolescence can be a tumultuous transitional period because of the desire for autonomy and denial of the disease.[35] Not surprisingly, the adolescents in this study also mentioned they could manage their asthma by themselves. In addition, the adolescents stated that they found it rather annoying and patronizing when their mothers continually reminded them to take their medication. It is interesting to consider whether these interferences of the mothers have a contrary effect and perhaps even become a reason for non-adherence in adolescents. The younger children however, expressed that their reason for taking their ICSs daily is that their parents told them to do so. It is known that asthma management in young children is highly dependent on parents.[36] Young children are unable to plan their own treatment (e.g. realising that they will need a bronchodilator before playing outside) and cannot have the primary responsibility for avoiding triggers (for example keeping their room dust-free).[37]

The strength of the focus group interviews in this study is that they build on the direct experiences of participants and allow for open-ended generation of ideas. It is a hypothesis-generating technique. Rather than testing a predetermined hypothesis, findings are gradually developed from the collected data and are continuously evolving and being tested against the existing data.[38] However, due to the fact that both the non-Dutch mothers and all children sometimes found it difficult to engage in conversation, it became necessary to stimulate and guide them using yes or no questions and examples. If we had not used this method, however, we would have known less about these groups. Furthermore, as is the case for most qualitative methods, the number of participants in our study was small. Moreover, the adolescent groups consisted of boys only. Despite these limitations, the study yielded interesting results as possible differences between ethnic groups in beliefs towards asthma and asthma treatment. These could be considered as hypotheses which should be further tested in quantitative studies.

## Conclusion

Consistent with earlier studies of patients' beliefs on asthma and practices in other populations, this study also showed that mothers and children have their own EMs of asthma and practices. Although ethnic differences were observed, the similarities between the mothers' and children's beliefs in this multi-ethnic population were striking. In particular, the fact that asthma is not seen as a chronic disease which requires daily use of an ICS, but as an acute phenomenon triggered by various factors, may be extremely relevant for clinical practice. It might suggest an explanation for some non-adherent behaviour. Beliefs about asthma and its treatment as seen in this study can be addressed during patient-clinician interactions. More specifically, a good understanding of the mothers' and children's beliefs about the illness and treatment is an important aspect in the management of asthma and will be a basis for good clinician-patient/family partnership in asthma care. In addition, in our multicultural society, health care providers should keep an open mind regarding consultations, especially with ethnic minority patients. It is also important for health care providers to reflect on the way differences in cultural values may play an important role in intercultural medical communication.

## Competing interests

The authors declare that they have no competing interests.

## Authors' contributions

QMvD has made contribution to acquisition of data, performed analysis and interpretation of data and have been involved in drafting the manuscript. KS participated in the design of the study, performed the analysis and interpretation of data and have been involved in drafting the manuscript. PJEB, FGO, JB and WMCvA participated in the design of the study, and have been involved in drafting the manuscript. The PEACE study group have made contribution to acquisition of participants. All authors read and approved the final manuscript.

## Pre-publication history

The pre-publication history for this paper can be accessed here:


